# SAC3D1: a novel prognostic marker in hepatocellular carcinoma

**DOI:** 10.1038/s41598-018-34129-9

**Published:** 2018-10-23

**Authors:** Myoung-Eun Han, Ji-Young Kim, Ga Hyun Kim, Si Young Park, Yun Hak Kim, Sae-Ock Oh

**Affiliations:** 1 Department of Anatomy, School of Medicine, Pusan National University, Yangsan, Republic of Korea; 2 BEER, Busan society of Evidence-based mEdicine and Research, Busan, Republic of Korea

## Abstract

Centrosome-associated proteins are recognized as prognostic factors in many cancers because centrosomes are critical structures for the cell cycle progression and genomic stability. SAC3D1, however, is associated with centrosome abnormality, although its prognostic potential has not been evaluated in hepatocellular carcinoma (HCC). In this study, 3 independent cohorts (GSE10186, n = 80; TCGA, n = 330 and ICGC, n = 237) were used to assess SAC3D1 as a biomarker, which demonstrated SAC3D1 overexpression in HCC tissues when compared to the matched normal tissues. Kaplan-Meier survival analysis also showed that its overexpression was associated with poor prognosis of HCC with good discriminative ability in 3 independent cohorts (GSE10186, *P* = 0.00469; TCGA, *P* = 0.0000413 and ICGC, *P* = 0.0000114). Analysis of the C-indices and AUC values further supported its discriminative ability. Finally, multivariate analysis confirmed its prognostic significance (GSE10186, *P* = 0.00695; TCGA, *P* = 0.0000289 and ICGC, *P* = 0.0000651). These results suggest a potential of SAC3D1 as a biomarker for HCC.

## Introduction

Centrosomal abnormalities are among the most important features of cancer cells because centrosomes are crucial for cell cycle progression and maintenance of genome stability^1,2^. Precise duplication of the centrosome and formation of the spindle apparatus during cell cycle guarantees the exact segregation of chromosomes. The centrosome consists of 2 centrioles surrounded by pericentriolar materials. The formation of the spindle apparatus from the centrosome involves an extensive rearrangement of microtubules. Its polymerization and depolymerization are tightly regulated by various molecules such as Microtubule Associated Protein 2 (MAP2)^3,4^, Microtubule Associated Protein 4 (MAP4)^5,6^, and Stathmin 1 (STMN1)^7^. Anticancer-drugs targeting microtubules have been developed because of reports on its role in tumorigenesis^1,2^.

*SAC3D1* (SAC3 homology domain-containing protein 1), a mammalian homologue of the *SAC3* (Saccharomyces suppressor of actin 3) gene in yeast, was discovered during a genetic screening procedure that sought out actin-associated genes^8^. It contains a Sac3 homologous domain in the middle region and 2 LXXLL motifs, which are signature motifs for transcriptional coactivators^9^. The *SAC3D1* gene is located at the chromosome 11 and its important paralog is MCM3AP (minichromosome maintenance complex component 3 associated protein)^10,11^. SAC3D1 is expressed in various tissues, including the liver and kidney. Interestingly, developing mouse embryos show its expression from E11.0 and it is higher in testes than in any other tissues^9^. A previous study suggested its role during cell cycle, centrosome duplication and spindle formation^12,13^. SAC3D1 is upregulated in inflammatory status in synovial tissues in patients with osteoarthritis compared to healthy control^14^. However, its role and significance in cancers remain poorly characterized.

The development of new drugs for hepatocellular carcinoma (HCC) is confronted with many challenges, even though HCC is the second-most common cause of cancer-related death worldwide^15–17^. Following the approval of sorafenib, several clinical trials have not yet shown successful results. Tyrosine kinase inhibitors with anti-angiogenic properties have only shown modest effects in treating HCC^18^. Several reasons such as comorbid cirrhosis and heterogeneous histological features and clinical factors have been suggested to explain poor results. However, detailed analysis of the clinical trials suggests new approaches. For instance, the REACH trial that used ramucirumab, which binds to the vascular endothelial growth factor receptor-2, on 565 patients was not noticeably sucessful^19,20^. However, survival benefits were observed in the patients with baseline serum AFP levels ≥400 ng/mL. Another example shows that although everolimus, an allosteric mTORC1 inhibitor, was unsuccessful in 546 patients during its phase III clinical trial^21^, and subsequent studies indicated that the loss of tuberous sclerosis complex2 (TSC2) was a strong predictor for HCC sensitivity to everolimus^22^. These analyses suggest that biomarker are crucial for the development of new drugs in treating HCC.

In this study, we examined the prognostic significance of SAC3D1 in HCC using three cohorts (The Cancer Genome Atlas (TCGA)^23,24^, the International Cancer Genome Consortium (ICGC)^25^, and the NCBI Gene Expression Omnibus (GEO) Series (GSE10186)^26,27^. The statistical analysis suggested it to be an important prognostic marker in HCC.

## Results

To evaluate the prognostic significance of SAC3D1 using public data-bases, we examined the information of 647 patients from 3 independent cohorts (GSE10186, n = 80; TCGA, n = 330 and ICGC, n = 237). Although the ICGC data did not provide subgroup information, the GSE10186 and TCGA data did. Out of 410 patients from the 2 databases, 150 patients showed alcohol use, 117 had hepatitis B, and 108 had hepatitis C. Patient information used in the present study is detailed in Table 1.

**Table 1:** Patients’ information used in current research in the GSE10186, TCGA and ICGC cohorts.

		GSE10186 (n = 80)	TCGA (n = 330)	ICGC (n = 237)
Stage	I	—	165	—
	II	—	80	—
	III	—	82	—
	IV	—	3	—
Subgroups	Alcohol consumption	47	103	—
	Hepatitis B	19	98	—
	Hepatitis C	58	50	—

### Overexpression of SAC3D1 in HCC

**Figure 1:**
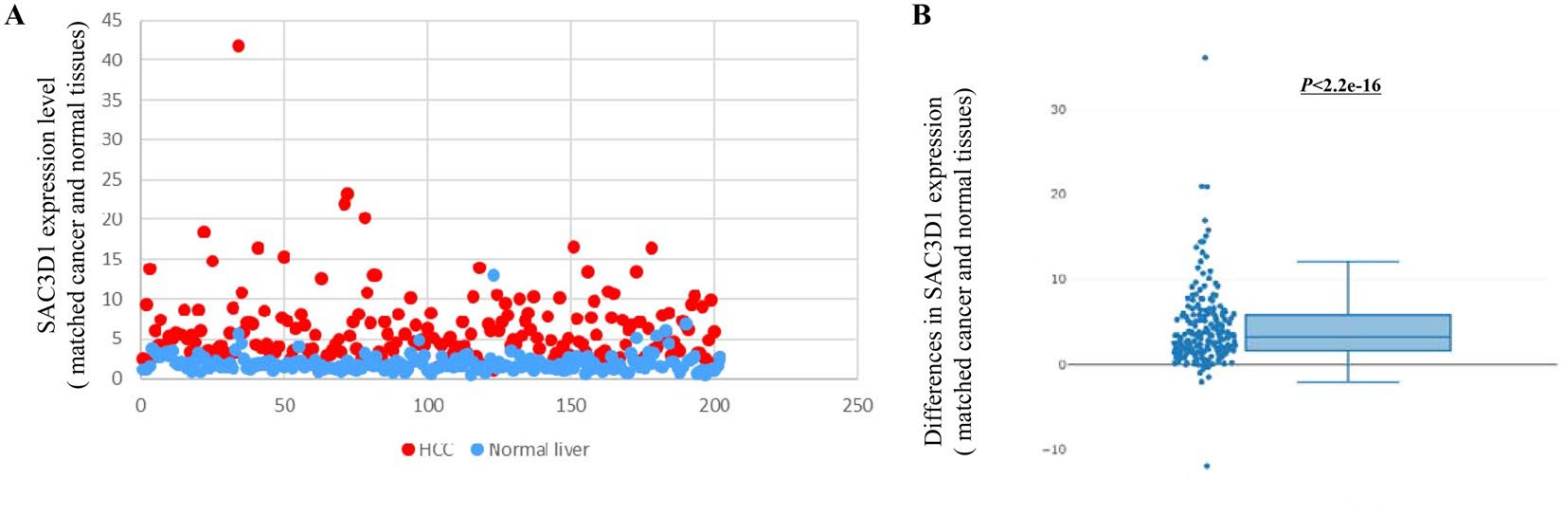
Comparison of SAC3D1 gene expression between cancer and matched normal liver tissues in the ICGC cohort. (**A**) The SAC3D1 expression levels in HCC and matched normal liver tissues. (**B**) The difference in SAC3D1 expression values between HCC and matched normal liver tissues. The scatters on the graph are values obtained by subtracting normal SAC3D1 expression from cancer SAC3D1 expression.

To evaluate the expression status of SAC3D1 in HCC, we compared its expression levels in cancer and matched normal liver tissues using ICGC data. SAC3D1 expression levels were observed to be significantly higher in cancer tissues than in their matched normal tissues (Fig. 1A; Table 2). In the Wilcoxon signed-rank test, HCC exhibited significantly higher SAC3D1 expression values than those by paired normal liver tissues (Fig. 1B).

**Table 2:** Comparison of SAC3D1 expression values between HCC and matched normal liver tissues.

		HCC	Normal liver
SAC3D1 expression value	Minimum	0.8808	0.4274
	1^st^ quarter	3.3515	1.2910
	Median	5.0427	1.6796
	3^rd^ quarter	7.5542	2.2805
	Maximum	41.8287	13.0589

### Prognostic significance of SAC3D1 in HCC

**Figure 2:**
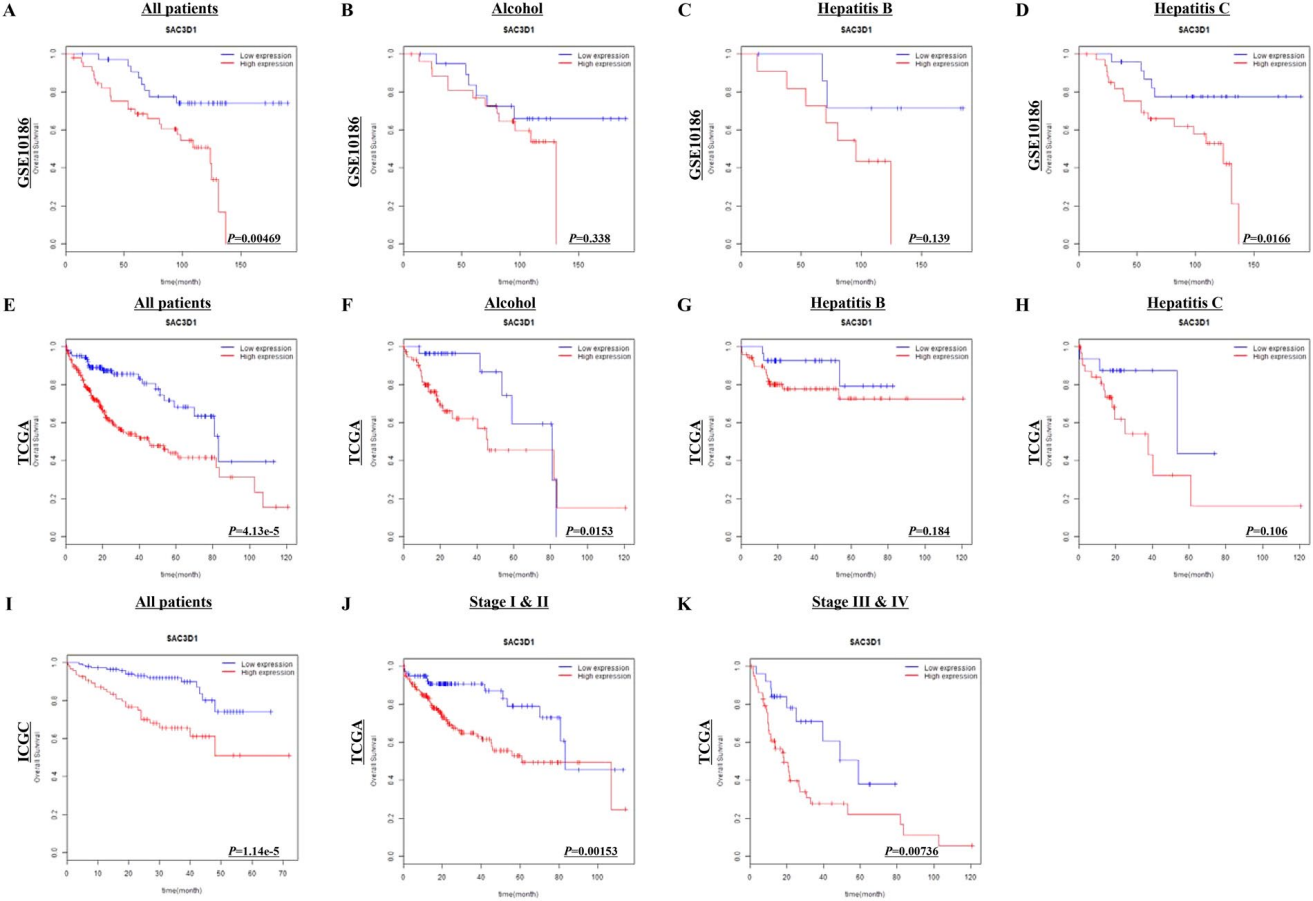
Kaplan-Meier survival analysis of HCC patients with respect to SAC3D1 gene expression. Overall survival in the GSE10186 (**A–D**), TCGA (**E–H**,**J**,**K**), and ICGC (**I**) cohorts were examined with respect to SAC3D1 gene expression. Survival analysis was performed for all the patients in each cohort (**A,E,I**) or for each subgroup of patients in each cohort. *P*-value was calculated using the log-rank test and is provided at the bottom right of each graph.

To evaluate the prognostic significance of SAC3D1, we examined the Kaplan-Meier curves for survival with respect to the SAC3D1 gene expression. Notably, higher expression of SAC3D1 showed shorter survival rates in the GSE10186, TCGA, and ICGC cohorts (*P* = 0.00469, *P* = 0.0000413, and *P* = 0.0000114, respectively; Fig. 2A,E and I). Moreover, SAC3D1 showed good predictive power in patients that consumed alcohol (*P* = 0.015) in the TCGA subgroup data analysis, but not in the GSE10186 subgroup data analysis (Fig. 2B,F). This predictive power was also demonstrated in earlier and later clinical stages of patients (*P* = 0.00153 and *P* = 0.00736, respectively; Fig. 2J,K). Furthermore, its prognostic significance was also shown in the multivariate analysis of the GSE10186, TCGA, and ICGC cohorts (*P* = 0.00695, *P* = 0.0000289, and *P* = 0.0000651, respectively; Table 3). SAC3D1 hazard ratio (HR) is particularly high when compared to the other factors (Table 3). As shown in Table 3 and Supplementary Fig. S1A, hepatitis B HCC patients have better outcome compared to non-hepatitis B HCC patients. In order to find the reason, pearson’s chi-square test was used to test differences in cancer stage between hepatitis B positive and negative patients. There were statistically significant differences of cancer stage between the groups (Supplementary Fig. S1B, χ^2^ = 22.719, df = 2, *P* = 1.1662e-05). SAC3D1 has also good discriminatory power in AFP-negative (<10 ng/ml) or TNM I stage HCC patients (Supplementary Fig. S2).

**Table 3:** Univariate and multivariate analysis of overall survival in each cohort (*, **, *** indicate significance at the <0.05, <0.01, <0.001).

Parameters	Univariate analysis				Multivariate analysis			
	P	HR	95 Cl		P	HR	95 Cl	
**GSE10186**								
Alcohol	0.797	1.097	0.4513	1.842	—	—	—	—
Hepatitis B	0.712	1.156	0.5347	2.501	—	—	—	—
Hepatitis C	0.859	0.9341	0.4411	1.978	—	—	—	—
SAC3D1	0.00739**	2.9517	1.337	6.516	0.00695**	3.0229	1.3539	6.7490
**TCGA**								
Age	0.189	0.9903	0.9952	1.025	—	—	—	—
Stage (I, II vs III, IV)	3.03e-6***	2.4341	1.675	3.536	9.92e-5***	2.1853	1.4742	3.2394
Gender (female vs male)	0.212	0.786	0.5385	1.147	—	—	—	—
Histologic grade (G1, 2 vs G3, 4)	0.674	1.084	0.7439	1.580	—	—	—	—
SAC3D1	0.0001***	2.5158	1.578	4.011	2.89e-5***	2.7560	1.7137	4.4323
Alcohol consumption	0.691	0.9194	0.6078	1.391	—	—	—	—
Hepatitis B	4.95e-5***	0.3515	0.2121	0.5824	9.35e-5***	0.3256	0.1855	0.5717
Hepatitis C	0.628	1.136	0.6773	1.907	—	—	—	—
**ICGC**								
Age	0.918	1.002	0.9714	1.033	—	—	—	—
Stage (I, II vs III, IV)	0.0085**	2.249	1.23	4.111	0.012*	2.8855	1.5174	5.4869
Gender (female vs male)	0.282*	0.4980	0.2671	0.9284	0.0053**	0.3871	0.1986	0.7543
SAC3D1	5.61e-5***	3.646	1.943	6.842	6.51e-5***	3.6307	1.9282	6.8364

### Biomarker performance of SAC3D1 in HCC

**Figure 3:**
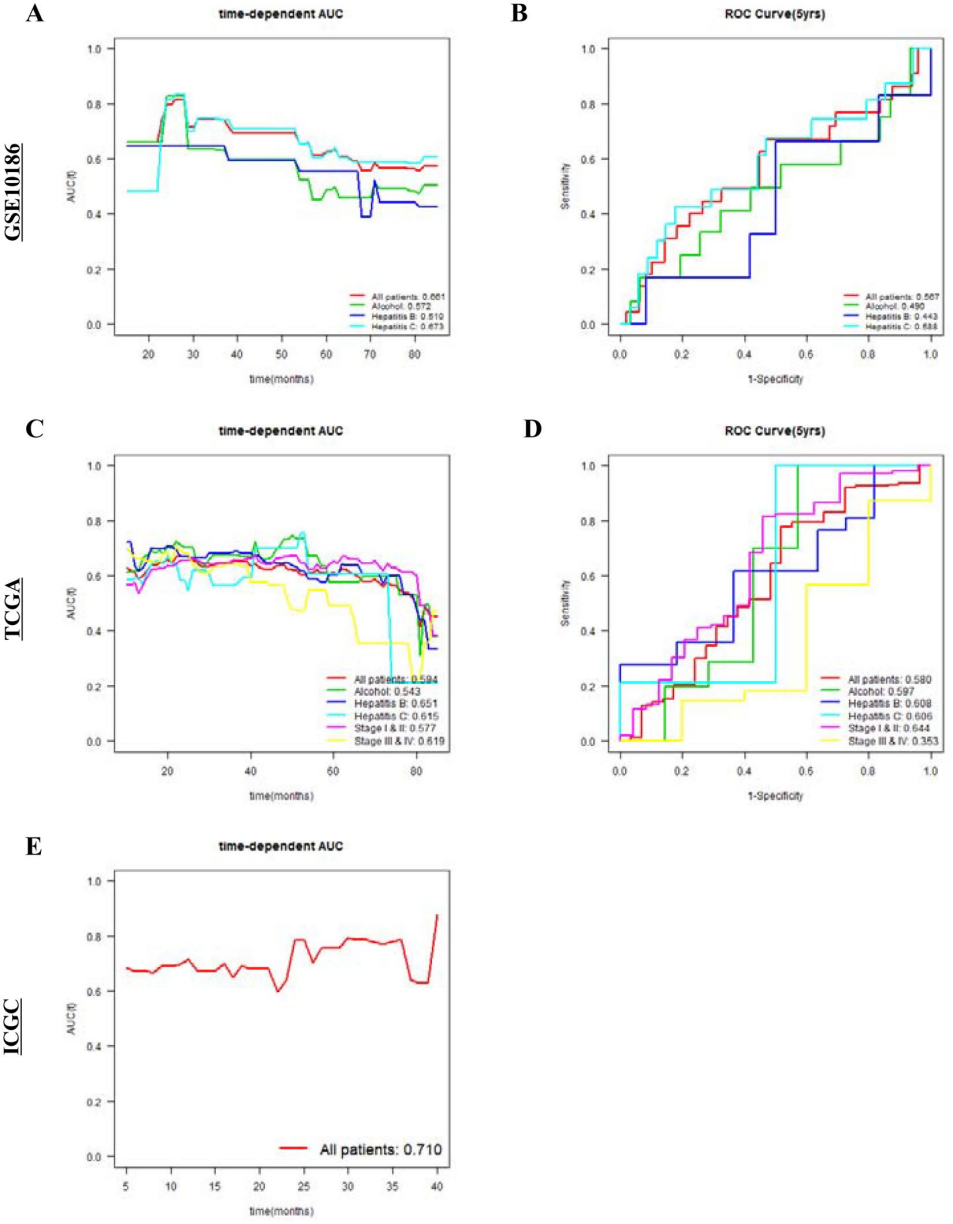
Time-dependent area under the curve (AUC) analysis and receiver operating characteristic (ROC) curves at 5 years with respect to SAC3D1 gene expression in the GSE10186, TCGA, and ICGC cohorts. Time-dependent AUC analysis and ROC curves at 5 years in the GSE10186 (**A**,**B**), TCGA (**C**,**D**), and ICGC (**E**) cohorts with respect to SAC3D1 gene expression (red: all patients, green: alcohol consuming patients, blue: Hepatitis B patients, and light blue: Hepatitis C patients). C-index values are shown at the bottom right of graphs (**A** and **C**). AUC values at 5 years are shown at the bottom right of graphs (**B** and **D**).

To assess the performance of SAC3D1 as a biomarker, we examined the Uno’s C-index values in the time-dependent Area Under the Curve (AUC) analysis and AUC values in the receiver operating characteristic (ROC) curves. For the comparison, we included well-known prognostic genes such as *BIRC5*, *CD34*, *GPC3*, *MK167*, and *TP53* in the analysis. SAC3D1 exhibited high C-index values for 5 years in 3 independent cohorts (GSE10186, 0.661; TCGA, 0.594; and ICGC, 0.710; Fig. 3A,C,E; Table 4). In the GSE10186 subgroup data analysis, SAC3D1 showed high C-index values in patients with hepatitis C (0.673; Table 4). Similar analysis of the TCGA data also showed high C-index values in patients with hepatitis B and C (0.651 and 0.615, respectively; Table 4). The 5-year AUC values showed a consistent pattern in the GSE10186 and TCGA cohorts (Table 5).

**Table 4:** C-index values of the specified parameters with respect to SAC3D1 and other established prognostic gene expressions in the GSE10186, TCGA, and ICGC cohorts.

Gene name	C-index (GSE10186)					
	All	Alcohol	Hepatitis B	Hepatitis C		
*SAC3D1*	0.661	0.572	0.510	0.673		
*BIRC5*	0.619	0.524	0.440	0.668		
*CD34*	0.545	0.565	0.461	0.537		
*GPC3*	0.510	0.507	0.643	0.394		
*MKI67*	0.404	0.374	0.353	0.358		
*TP53*	0.361	0.255	0.181	0.346		
**Gene name**	**C-index (TCGA)**					
	**All**	**Alcohol**	**Hepatitis B**	**Hepatitis C**	**Stage I & II**	**Stage III & IV**
*SAC3D1*	0.594	0.543	0.651	0.615	0.577	0.619
*BIRC5*	0.646	0.518	0.595	0.532	0.590	0.705
*CD34*	0.375	0.561	0.461	0.568	0.374	0.420
*GPC3*	0.516	0.552	0.531	0.530	0.507	0.605
*MKI67*	0.637	0.438	0.481	0.536	0.615	0.605
*TP53*	0.457	0.566	0.390	0.511	0.401	0.589
**Gene name**	**C-index (ICGC)**					
	**All**					
*SAC3D1*	0.710					
*BIRC5*	0.766					
*CD34*	0.425					
*GPC3*	0.608					
*MKI67*	0.707					
*TP53*	0.461

**Table 5:** AUC values at 5 years for the specified parameters with respect to each gene in the GSE10186 and TCGA cohorts.

Gene name	AUC value at 5 years (GSE10186)					
	All	Alcohol	Hepatitis B	Hepatitis C		
*SAC3D1*	0.567	0.490	0.443	0.588		
*BIRC5*	0.517	0.544	0.443	0.526		
*CD34*	0.534	0.501	0.454	0.550		
*GPC3*	0.484	0.539	0.622	0.391		
*MKI67*	0.434	0.488	0.433	0.385		
*TP53*	0.334	0.239	0.210	0.331		
**Gene name**	**AUC value at 5 years (TCGA)**					
	**All**	**Alcohol**	**Hepatitis B**	**Hepatitis C**	**Stage I & II**	**Stage III & IV**
*SAC3D1*	0.580	0.597	0.608	0.606	0.644	0.353
*BIRC5*	0.627	0.603	0.542	0.500	0.651	0.474
*CD34*	0.386	0.521	0.435	0.847	0.395	0.435
*GPC3*	0.536	0.605	0.260	0.392	0.572	0.580
*MKI67*	0.586	0.465	0.423	0.419	0.624	0.410
*TP53*	0.528	0.540	0.463	0.622	0.520	0.606

## Discussion

The centrosome is crucial for cell cycle progression and maintenance of genome stability. In the present study, we showed the prognostic importance of SAC3D1, which has been associated with the formation of the centrosome and spindle apparatus^12,13^. Its prognostic significance was also validated in 3 different kinds of HCC cohorts. The results suggest the potential of SAC3D1 as a biomarker for HCC.

Several genes associated with the formation of the centrosome and spindle apparatus are also associated with the prognosis of HCC. Aurora A kinase, a good prognostic marker for HCC, is a serine/threonine kinase that is required for the recruitment of several proteins such as the transforming acidic coiled coil (TACC) family of proteins and kinesin 5 (crucial for spindle formation)^28,29^. Its overexpression is associated with high-grade and high-stage HCC^30^. Another example is TPX2, which is necessary for microtubule nucleation in a RanGTP-dependent manner^31,32^. RNAi-mediated knockdown of *TPX2* inhibits spindle fibre formation in HeLa cells^33^ and cell proliferation and viability in Hep3B and HepG2 hepatic cancer cells^34^. It also regulates Aurora kinases during mitosis^35^. In consistent with previous results, SAC3D1, which associated with the formation of the centrosome, is overexpressed in HCC compared to matched normal tissue.

Earlier studies about SAC3D1 in yeasts and mammalian cells suggests that it plays a role in the cell cycle^12,13^. In the original yeast screen study, 5 related genes (*SAC1*–5) were identified and aberrant organization of intracellular actin and deposition of chitin at the surface were observed in the mutants^8^. In the later yeast study, the SAC3 protein was suggested to reside in the nucleus and was associated with the progression of the cell cycle^12^. SAC3 mutants also showed a delay in the G2/M phase. Moreover, the mutants showed diverse abnormalities in the spindle morphology, including short misoriented spindles in large-budded cells and elongated spindles. A study in mammalian cells showed roles of SAC3D1 in mitotic progression, centrosome assembly, and spindle assembly^13^. It was located in the mitotic structure and siRNA-transfected SAC3D1 cells showed one centrosome and an increased number of micronuclei during mitosis. In contrast, SAC3D1-GFP transfected cells showed centrosome amplification and multiple spindle poles. These roles of SAC3D1 during the cell cycle support its prognostic significance in hepatic and renal cancers. In the current study, we found that the higher the SAC3D1 expression level in all three cohorts, the worse the patient’s prognosis. However, in the subgroup analysis, alcohol consumption and hepatitis B patients in GSE10186 and hepatitis B, C groups in TCGA were not statistically significant. Since subgroup analysis has a smaller number of patients than the total number of patients, obtaining statistically significant results can be difficult. Therefore, our subgroup analysis should pay attention to the tendency that high SAC3D1 expression is associated with bad prognosis, not statistical significance. Some studies reported that patients with hepatitis B have better prognosis compared to patients with hepatitis C virus^36,37^. The TCGA analysis in Table 3 shows interesting results. HCC patients with hepatitis B as a risk factor have better outcome than other HCC risk factors. The exact reason seems to require a lot of research, but the results are similar to those of other researchers.

Unexpectedly, SAC3D1 knockout mice did not show any defects in their growth^9^. Mutant mice were grossly normal, and no proliferation defects were reported. It is possible that another Sac3 homology domain-containing protein called MCM3AP, which is essential in initiating DNA replication, compensated for the loss of SAC3D1 in these mutant mice^10,11^.

Because predicting prognosis of cancer patients is critical for therapeutic decisions, many researchers have been developed several prognostic factors in many cancer types including HCC^38–40^. Among many studies, Oncotype DX predicts the prognosis of breast cancer patients with 21 mRNA levels, not the protein level in actual clinical practice^40^. Although research at the protein level is essential for studying the function of SAC3D1, mRNA level studies are effective in predicting prognosis. In the current study, we found that the higher the SAC3D1 expression level in all three cohorts, the worse the patient’s prognosis. However, in the subgroup analysis, alcohol consumption and hepatitis B patients in GSE10186 and hepatitis B, C groups in TCGA were not statistically significant. Since subgroup analysis has a smaller number of patients than the total number of patients, obtaining statistically significant results can be difficult. Therefore, it is important that the subgroup analysis results show that the higher the SAC3D1 expression level is, the statistically significant, the worse the patient’s prognosis. An interesting aspect of our study is that the prognosis of hepatitis B HCC patients is better than that of non-hepatitis B patients. There is no detailed information such as drug use data for patients is unknown, but it was found that the cancer stage of hepatitis B patients was lower than that of non-hepatitis B patients. These results are consistent with the results of other researches^36,37^. Although there are currently limitations in mRNA-based study of SAC3D1, we are sure that it is sufficient to suggest the possibility of it as a prognostic gene for HCC.

## Methods

### Data acquisition and characteristics

The primary and processed data were downloaded from GSE10186^26,27^, TCGA^23,24^ and, ICGC^25^ in December 2017. We obtained the mRNA expression data and clinical information as detailed in the Supplementary Files 1–3. The following samples were excluded: (1) “0” gene expression values and (2) insufficient survival information. These processes were performed using the R statistical software with the help of the “*cgdsr*” and “*GEOquery*” packages (Supplementary Files 4–6).

### Wilcoxon signed rank test

The differences of SAC3D1 expression between cancer and matched normal liver were not a normal distribution and therefore Wilcoxon signed rank test was performed. The Wilcoxon signed-rank test was performed to analyse the SAC3D1 expression values between paired cancer and normal liver samples using the “*coin*” package.

### Survival analysis

Survival analyses were performed to predict the overall survival (OS). Kaplan-Meier survival curves were used to evaluate the accuracy of the discrimination. In the Kaplan-Meier analyses, we determined the optimal cut-off value that had the maximal Uno’s C-index using a 5-fold cross-validation. Furthermore, we used 2 methods to evaluate biomarker performance: [1] Uno’s C-index in the time-dependent area under the curve (AUC) analysis and [2] AUC values in receiver operating characteristic (ROC) curves at the 5 year mark. These values were obtained using the R packages “*survival*” and “*survAUC*”. C-indices and AUC values of 0.75 or greater were considered to have excellent predictive value, and values of 0.6 or greater were considered acceptable for survival predictions. We used univariate and multivariate Cox regression analyses to compare the effect of SAC3D1 on prognosis along with other clinical variables. In multivariate analysis, we included clinical variables that are not related to survival in the univariate analysis for considering the confounders. We did not describe the values that are not significant in the multivariate cox regression results in Table 3. Statistical analyses were performed using the R software version 3.5.0 (The R Foundation for Statistical Computing, 2018).

## Electronic supplementary material

Supplementary information

## Data Availability

All data generated or analysed during this study are included in this published article (and its Supplementary Information files).
