# Supplementary material for: SAC3D1: a novel prognostic marker in hepatocellular carcinoma
Source: Sci Rep. 2018 Oct 23;8:15608. doi: 10.1038/s41598-018-34129-9 (PMC6199250; doi:10.1038/s41598-018-34129-9)
Supplement: Supplementary file 1 — Supplementary information [file 41598_2018_34129_MOESM1_ESM.pdf]

SAC3D1: a novel prognostic marker in hepatocellular carcinoma

Myeong-Eun Han[1], Ji-Young Kim[1], Ga Hyun Kim[1], Si Young Park[1], Yun Hak Kim[1,2,*], Sae-Ock Oh[1,*]

[1]Department of Anatomy, School of Medicine, Pusan National University, Yangsan, Republic of Korea

[2]BEER, Busan society of Evidence-based mEdicine and Research, Busan, Republic of Korea

Myeong-Eun Han and Ji-Young Kim contributed equally to this work.

Figures: 3

Tables: 5

Corresponding authors:

Yun Hak Kim, MD, PhD.

Department of Anatomy, School of Medicine, Pusan National University,

Yangsan, Gyeongnam, Republic of Korea

Tel: +82-51-510-8091, Fax: +82-51-510-8049

E-mail: yunhak10510@pusan.ac.kr

Sae-Ock Oh, MD, PhD.

Department of Anatomy, School of Medicine, Pusan National University,

Yangsan, Gyeongnam, Republic of Korea

Tel: +82-51-510-8045, Fax: +82-51-510-8049

E-mail: hedgehog@pusan.ac.kr

Supplementary Information

Supplementary Figure S1. Comparison of overall survival between hepatitis B and non-hepatitis B HCC patients. (A) Kaplan-Meier survival curve of HCC patients with respect to hepatitis B virus infection. (B) Differences of cancer stage between hepatitis B and non-hepatitis B HCC patients. HBV (-) or (+) means no infection or infection of hepatitis B virus. Relative percent = [HBV (-) or (+) patients in given stage] / [Total HCC patients in given stage].

Supplementary Figure S2. Kaplan-Meier survival analysis of HCC patients with respect to SAC3D1 gene expression. Survival analysis was performed for each subgroup of patients in TCGA cohort. P-value was calculated using the log-rank test and is provided at the bottom right of each graph.

Supplementary Figure S3. Expression levels of SAC3D1 in patients with HCC (GSE20238). 0 means no vascular invasion, and 1 means vascular invasion.

Supplementary Table S1. Optimal cutoff values of genes in each cohort.

Supplementary Table S2. Patients' information of used in current research in the GSE20238.

Supplementary File 1. Data combining the clinical and genetic information of the GSE10186 cohort (R data).

Supplementary File 2. Data combining the clinical and genetic information of the TCGA cohort (R data).

Supplementary File 3. Data combining the clinical and genetic information of the ICGC cohort (R data).

Supplementary File 4. R code for the GSE10186 cohort.

Supplementary File 5. R code for the TCGA cohort.

Supplementary File 6. R code for the ICGC cohort.

Supplementary Figure S1

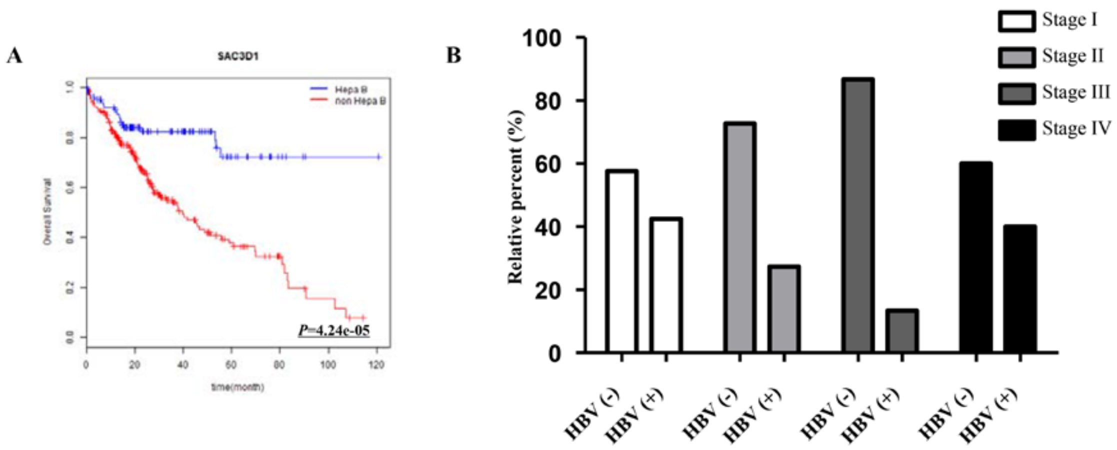

Supplementary Figure S2

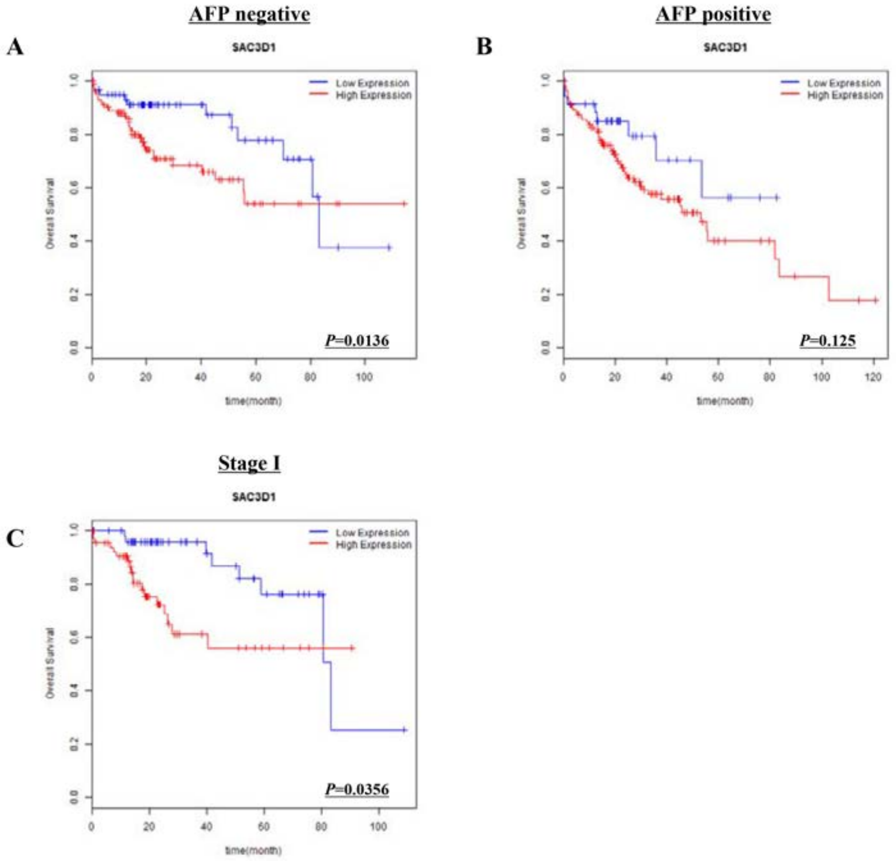

Supplementary Figure S3

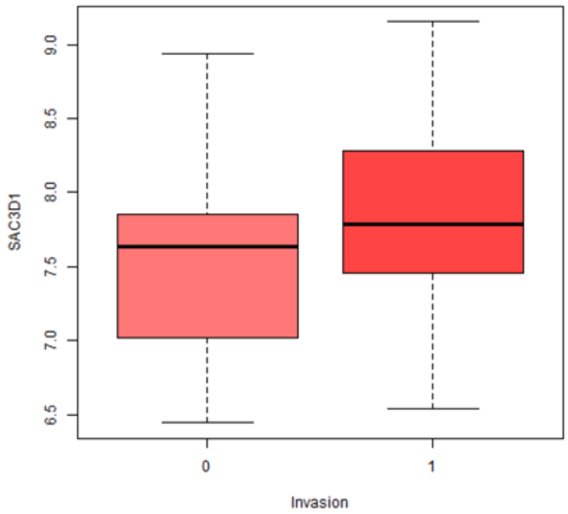

Supplementary Tables

**Gene name**	**Cutoff values**		
	**GSE10186**	**TCGA**	**ICGC**
SAC3D1	**4485.109**	**286.1798**	**7.218211**
BIRC5	**15929.74**	**288.3768**	**5.3804**
CD34	**4386.906**	**1306.763**	**8.24675**
GPC3	**12135.64**	**5818.894**	**229.1579**
MKI67	**628.5131**	**1073.798**	**2.956712**
TP53	**2035.01**	**632.0375**	**5.44815**

Supplementary Table S1. Optimal cutoff values of genes in each cohort.

	Vascular invasion		BCLC staging			
	**No (0)**	**Yes (1)**	**0**	**A**	**B**	**C**
**# of patients**	34	45	9	56	7	8

Supplementary Table S2. Patients' information of used in current research in the GSE20238.
